# Identifying hazards for psychological harm in cancer care using incident reports: searching for the invisible

**DOI:** 10.1093/intqhc/mzaf099

**Published:** 2025-11-04

**Authors:** Yvonne Pfeiffer, Lara Dreismann, Sofia C Zambrano, David L B Schwappach

**Affiliations:** Dr. Yvonne Pfeiffer, Harmfree Healthcare, Wädenswil, Switzerland; Faculty of Medicine, Institute of Nursing Science (INS), University of Basel, Basel, Switzerland; Institute of Social and Preventive Medicine (ISPM), University of Bern, Bern, Switzerland; Institute of Social and Preventive Medicine (ISPM), University of Bern, Bern, Switzerland; Institute of Social and Preventive Medicine (ISPM), University of Bern, Bern, Switzerland

**Keywords:** incident reporting and analysis, hazard identification, risk assessment, psychological harm, underreporting

## Abstract

**Background:**

Keeping patients safe means not only preventing physical harm events, but also psychological harm. However, little is known about psychological harm in health care. The aim of this study was to investigate whether and what types of hazards for preventable psychological harm (PPH) were present in incidents reported by cancer care healthcare workers.

**Methods:**

Incidents reported by staff working in the wards and ambulatory infusion centres providing cancer care in three Swiss hospitals during the years 2022 and 2023 were qualitatively analysed in order to identify hazards for psychological harm. In an iterative process *N* = 623 reports were explored and a coding scheme developed.

**Results:**

The generated coding scheme distinguished events solely involving primary PPH from events in which primary PPH co-occurred with physical harm. Two and a half percent (2.5%) of the reports pointed to hazards for primary PPH, and 15% (15.4%) of the incidents reported PPH co-occurring with physical harm. The most frequently identified PPH hazard was ‘not providing appropriate information to patients and their close others’. Events that were potentially psychologically harmful included ‘communication issues’, ‘privacy or autonomy violations’, and ‘poor transitions of care’. In the group of incidents in which the potential for physical and psychological harm were concurrently prevalent, we identified errors potentially affecting the effectiveness of cancer care, and instances in which patients or their close others detected or prevented an error. Relatives or close others were mentioned in 17 (18%) of the PPH-related reports.

**Conclusion:**

This study suggests that while incident databases do contain reported hazards for primary PPH, it is likely that they are largely under represented. In line with existing research, we found hazards for PPH such as privacy or autonomy violations. Well-known safety hotspots, such as patient transfers, may pose hazards for psychological harm, suggesting that designing strong organizational practices may help prevent psychological harm. Future research needs to use other data sources and shed light on various perspectives to learn more about the variety of PPH, its causes, and potential consequences and preventive measures.

## Introduction

Adverse events in healthcare are defined as negative health outcomes that do not stem from the disease of the patient but are a consequence of the health care received. Reducing the occurrence of ‘preventable’ adverse events is the core aim of patient safety improvement activities [1]. Traditionally, these activities focus on decreasing preventable physical harm to patients, such as healthcare-associated infections. However, harm in healthcare extends beyond just physical injury. For example the WHO expects patient safety efforts to reduce ‘death, disability, and physical and “psychological” injury from unsafe care’ [2, p.16].

Psychological harm is often discussed as a consequence from a physical harm event. For example, patients report poor disclosure practices in the aftermath of medical error to be harmful. However, preventable psychological harm (PPH) as an event itself remains rather understudied in patient safety [2]. The term ‘emotional harm’ is often used interchangeably [2–4]. It develops from a negative deviation from the ‘standard of patient centred care’ that poses the patient at risk for harm [5]. Prior research has reported a variety of psychologically harmful events [3, 6, 7] that are preventable, primarily in the context of disrespectful interactions: for example, being treated like an object or the patient’s autonomy being violated [6], experiencing disrespect, such as health care workers (HCW) not introducing themselves or patients being called ‘dear’ [7], or experiencing a violation of privacy [3].

The potential consequences of these harmful events are not only immediate negative emotions, but could also cause manifold longer-lasting effects such as loss of trust in healthcare providers, potentially leading to avoidance of necessary care in the future, or to sharing less information with providers [6]. In specifically vulnerable patient groups, for example at the end-of-life, poor communication itself is seen as inherently harmful by patients and families [8]. Accordingly, older adults were found to experience psychological harm in the emergency departments [4], and psychological harm was reported to occur in a radiology department [4]. While physical harm is frequent in cancer care [9, 10], the complexity of the illness and the intensity of treatments increase the potential for preventable psychological harm, making it a critical patient safety issue. While prior qualitative research suggests that the negative consequences of PPH are significant for patients and their families [2, 3, 6, 7, 11], there is no systematic empirical assessment, largely due to a lack of definitions and standardized measurements. Consequently, our understanding of PPH is still in its infancy.

That drawing up a wrong dose in a syringe represents an important hazard is well understood, even if the error is caught before administration and no actual harm occurs. In analogy to physical harm, hazards for psychological harm also warrant prevention activities, even if the patient ends up not being hurt, for example, if they did not hear a disrespectful comment. The criterion for determining whether the hazard for PPH was present is therefore not the actual consequence(s) for a specific patient, but rather its potential to cause harm to any patient. As part of a larger research project, we focus in this study on ‘primary’ preventable psychological harm–situations that are psychologically harmful ‘independent’ of any physical harm event, e.g. communicating a diagnosis to the wrong patient.

While psychological harm can result from a disease or its treatment (e.g. anxiety after experiencing pain), we concentrate on hazards for ‘preventable’ psychological harm events, i.e. those events that could be prevented if appropriate organizational measures were taken. While it may seem that the causation and thus preventability of psychological harm lies entirely in the actions of individual healthcare providers, there are powerful organizational practices and culture that shape how HCW interact with their patients and their families or close others. For example, the design of information flows affects how well-informed the HCW can be about a patient. Similarly, culture influences communication styles, which can be important in preventing psychological harm [12].

Incident reporting systems (IRS) are designed to identify unknown hazards in healthcare institutions by encouraging staff to report safety-relevant events. In contrast to many countries, in Switzerland, IRS target near-misses, i.e. events having the potential to injure patients, but where no actual harm occurred (see national guide on implementing IRS [13]). Despite this objective, some events were reported in which physical harm seems likely though no adverse outcomes were reported. The causal analysis of the reported events is expected to identify hazards related to the involved processes, structures, materials, and organizational or individual behaviours. From these analyses, systemic patient safety improvements should be designed and implemented [14]. Due to underreporting and reporting biases, incident reports cannot be used to evaluate the frequency or relative importance of certain events or hazards [15]. The causal analysis of reported incidents is restricted by the context and information that is provided in them. Despite these limitations, IRS reports reflect what HCWs consider reportable incidents. They thus are valuable in two regards: (i) identifying emerging hazards that are yet unknown and (ii) drawing conclusions about what is regarded as safety-relevant and reportable by HCW. As the reports give insights into hazards that may lead to patient harm, this study presents a prospective hazard analysis, identifying psychological harm that could occur under the reported circumstances. In conclusion, we were looking for unsafe (individual and organizational) actions or circumstances that may lead to psychological harm.

The aims of this study were (i) to investigate whether hazards for primary PPH were present in patient safety incidents reported by HCW providing cancer care; and (ii) to identify and categorize different types of PPH hazards. We focused on incidents reported by HCW caring for cancer patients, as this patient group is particularly vulnerable to experience preventable psychological harm. As psychological harm may also affect the patient’s close others [8, 16], we also aimed to assess whether close others were mentioned in the incident reports.

## Methods

### Data collection and sample

We obtained all incident reports from the wards, inpatient units, and outpatient treatment centres providing cancer care (*N* = 640) across three Swiss hospitals, including one university hospital, and two large cantonal hospitals. University and cantonal hospitals are the main providers of cancer care in Switzerland, and they are using IRS with the aim to learn from events and improving patient safety. The incident reports were submitted to the hospitals’ IRS during the years 2022 and 2023. After deleting duplicate reports, 623 reports were investigated for hazards for psychological harm. The study analysed incident reports that were anonymized by the participating hospitals and without a linkage to any patient-related databases. A cooperation agreement specifying the sharing of incident reports was established and approved between each hospital and the university’s research team. Participation of the hospitals was voluntary and without financial incentive. As the study involved fully anonymized Critical Incident Reporting data, it did not require approval of an ethical committee in Switzerland, in accordance with Articles 1 and 2 of the Federal Act on Research involving Human Beings (Human Research Act, HRA [17]).

### Research team

The research team consisted of four experienced researchers with the following backgrounds: researcher 1 is a postdoctoral researcher in patient safety, and researcher 2 is a postdoctoral psycho-oncologist, researchers 3 and 4 are the principal investigators of the research project, senior experts in end-of-life communication and patient safety, respectively.

### Data analysis and in-and exclusion criteria

**Figure 1:**
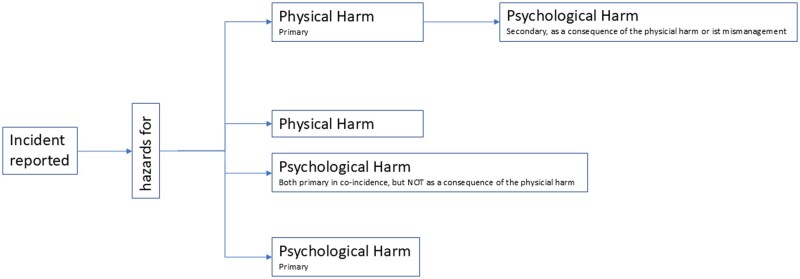
Differentiation of primary and concurrent PPH

The analytical approach was a prospective hazard identification. As the reports were the only data available, we could not apply a full hazard assessment technique [18, 19]. We assessed whether hazards for psychological harm were present in the reported situations (see Fig. 1) in evaluating them (i) against deviations from standard patient-centred care that may hurt a patient psychologically, i.e. giving a diagnosis to the wrong patient, and (ii) against potential psychological consequences they could have for the patient, such as loss of trust. Since the incidents do not provide information on whether and how much harm occurred, the severity of the identified potential psychological harm could not be evaluated. Accordingly, the mentioning of psychological sequelae of an event, for example, a distressed patient or relative crying was not a necessary precondition for classifying an event as potentially psychologically harmful. In contrast, reports describing situations focusing on physical harm that may have led to potential psychological harm as a consequence were not included in the analysis, as the focus was the assessment of psychological harm as an event in itself.

A definitory distinction was developed as first part of the coding scheme (see Fig. 1): reports that focused exclusively on situations of potential psychological harm, in which the psychological harm was the only event reported (called ‘primary’ PPH), were differentiated from reports that focused on situations in which psychological harm co-occurred in the context of a potential physical harm situation (called ‘concurrent’ PPH). All incidents potentially jeopardizing the effectiveness of the received cancer care were categorized as hazards for psychological harm (concurrent PPH), because errors in cancer treatment such as delayed or wrongly timed or dosed treatments can have irreversible health consequences and may therefore lead to major psychological harm.

Data analysis consisted of three major steps: First, all instances of hazards for preventable psychological harm were identified, and a coding scheme was iteratively developed. Second, the coding scheme was applied to all incidents. Third, the identified hazards for PPH were categorized.

In the first step, all reports were analysed by researcher 1 and unclear incidents were discussed between researchers 1 and 2. The whole team (see above) developed the above-mentioned distinctions. Researcher 1 and 2 developed the categories for describing different kinds of hazards for psychological harm, integrating feedback from researchers 3 and 4. In the second step, researcher 1 re-analysed the whole set of incidents, resolving unclear identifications of psychological harm with researcher 2. Then, researcher 2 also analysed a subset (*n* = 70, 11% of the sample). Two instances of non-alignment between the researchers’ codings were solved in discussion. Finally, the identified reports containing hazards for primary and concurrent harm were sorted into different categories in an iterative process between researchers 1 and 2 and the larger research team.

## Results

From the 623 analysable incident reports, we identified 16 (2.5%) that contained hazards potentially contributing to primary PPH. Eighty (12.8%) incidents reported hazards for concurrent PPH. Table 1 lists the number of analysed reports, as well as the frequency of primary and concurrent hazards identified across the three hospital sites.

**Table 1.** Sample description^a^

Sample	All reports^b^	Primary PPH reports^c^	Concurrent PPH reports^c^	All psych. harm reports^b^
No. of incidents hospital 1^c^	290 (46.5%)	6 (2.1%)	20 (6.9%)	26 (9%)
No. of incidents hospital 2^c^	227 (36.4%)	8 (3.5%)	31 (13.7%)	39 (17.1%)
No. of incidents hospital 3^c^	106 (17%)	2 (1.9%)	29 (27.4%)	31 (29.2%)
Total^b^	623 (100%)	16 (2.6%)	80 (12.8%)	96 (15.4%)

a PPH = preventable psychological harm.

b Percentages in columns proportion of total number of incidents analysed (*n* = 623).

c Percentages as proportion of total in institution, in row.

The types of PPH hazards differentiated in the coding scheme are listed in Tables 2 and 3, together with the number of reports they were identified in, alongside examples. The most frequently reported hazard of primary PPH, i.e. that was reported independent from potential physical harm, was related to ‘providing inappropriate or contradictory information to patients or their close others’ (*n* = 5, 31%), for example about subsequent cancer treatment (see Table 2). Experiencing or witnessing a ‘violation of privacy’ as well as situations in which the ‘autonomy’ of a patient was not respected were identified in three incident reports each as potential sources of primary PPH.

**Table 2.** Types of potential primary psychological harm identified in the analysed incident reports^a^

		Frequency^a^	Reported hazardous situations, examples		
**Type of primary preventable psychological harm**					
	Inappropriate/inexistent/contradictory patient/close other information/communication	5 (31%)	Not being adequately informed about cancer treatment before its start; the treatment plan handed to caregiver of patient does not correspond with actual treatment plan; contradictory communication to patient from different HCW		
	(Witnessing) privacy violation, breach of confidentiality	3 (19%)	Disclosing sensitive information (e.g. about diagnosis) to other patients, because patient list has been left in the open; accidentally being informed about another patient’s health issue or procedure because HCW misidentifies one patient for another		
	Disregarding the patient, their preferences and/or autonomy	3 (19%)	Being fed via gastric tube despite declared refusal; being prescribed with treatment despite patient’s refusal which is noted in EHR; not being greeted by a group of physicians walking by		
	Experiencing poor transitions (between wards or units, or poor discharge)	1 (6%)	Being sent to the wrong location for treatment, staff having a hard time finding out where patient needs to go		
	Failure to care for personal possessions	1 (6%)	List of personal items cannot be read		
	Mixed: Reported incidents that were not categorized as belonging to a type	3 (19%)	DNR status not noted in EHR chart; unnecessary isolation; rushed patient discharge from hospital room		
Total		16 (100%)			
**Reports comprising involvement of close others**					
	Close other (e.g. parent, partner) being mentioned	4 (25%*)	Wife having to advocate for removal of the gastric tube that was inserted against the patient’s declared will; parent identifying inconsistency between chemotherapy treatment plan handed to them and treatment plan in use at hospital; husband and patient not being informed properly about intervention		

a The percentage of reports from the number of reports identified as PPH (*n* = 16). HCW = healthcare worker; EHR = electronic health record. All examples are paraphrased to maintain anonymity of the participating institutions.

**Table 3.** Types of concurrent psychological harm identified in the incident reports

		Frequency^a^	Reported hazardous situations, examples		
**Type of concurrent preventable psychological harm**					
	Error potentially affecting the effectiveness of cancer care	22 (28%)	Missing radiotherapy sessions; chemotherapy that should be aligned with radiotherapy is not administered; dose of chemotherapy administered too low		
^b^	Experiencing poor transitions (between wards or units, or poor discharge)	15 (19%)	Patient not being informed and waiting in the wrong place for his appointment for radiotherapy; discharge papers are not provided in time, and patient has to leave without; nursing home not capable of caring for a patient’s central catheter he is discharged with.		
	Patient/close other detecting/preventing error	15 (19%)	HCW ask patients about meds or treatments because of incomplete documentation; parent realizing wrong chemotherapy doses were dispensed for home administration; close other noticing that chemotherapy was based on wrong weight and thus overdosed.		
	Not receiving appropriate care in emergency situation	6 (8%)	Patient not able to breathe due to blocked inner cannula that was not appropriately cleaned; patient being sent to get emergency CT-scan, but not being admitted there and sent back		
^b^	Inappropriate/inexistent/inconsistent patient/close other information/communication	5 (6%)	Parents not educated about handling of toxic drug waste; procedure cannot be performed because information to stop certain medication before hand was not given		
	Minimizing patient concerns	4 (5%)	Child’s complaints about pain are not taken seriously and only realized when swelling was detected during disconnecting infusion; physician not granting request to increase pain medication, explaining that the patient can manage		
	Being exposed to infection hazard due to mismanagement	3 (4%)	Staying in a wardroom with a patient who is not immediately isolated after being diagnosed with COVID		
	Witnessing time-consuming, cumbersome information gathering by HCW for own care	2 (3%)	Unclear and incomplete prescriptions, nurse needing to find out how drugs should be administered, and premedication is missing		
	Interacting with HCW that are not well informed about patient	2 (3%)	Patient’s chart being transferred from emergency department to the ward is over hours not accessible, HCW do not know the diagnoses or necessary procedures		
^b^	(Witnessing) privacy violation, breach of confidentiality	1 (1%)	Patient receiving another patient’s prescription		
^b^	Disregarding the patient, their preferences and/or autonomy	1 (1%)	Overriding patients’ concerns about medication (patient was right in the end)		
^b^	Failure to care for personal possessions	1 (1%)	Patient being administered a too high dose of a drug that is expensive and that they brought to the hospital		
Mixed: Reported incidents that were not categorized as belonging to a type group		3 (4%)	Receiving consultation/medication of other patient (2); unnecessary emergency care due to wrongly drawn blood sample		
Total		80 (100%)			
**Reports comprising involvement of close others**					
	Close other (e.g. parent, partner) being mentioned	13 (20%)	Parents administering the wrong drug to child at home due to wrong plan; wife calling emergency department because husband with leukaemia is febrile, her call is not treated as the emergency it is and contact to the haematology department denied		

a The percentage of reports from the number of reports identified as concurrent PPH (*n* = 80).

b Type of harm identified also in primary harm events. All examples are paraphrased to maintain anonymity of the participating institutions.

Three types of concurrent PPH hazards made up two thirds of all concurrent PPH reports (see Table 3): first, an ‘error potentially affecting the effectiveness of cancer care’; second, ‘experiencing poor transitions (within hospitals or between institutions)’; and third, situations in which a ‘patient or their close other were the ones to detect or prevent an error in the care’.

Five types of PPH were identified in primary as well as concurrent PPH hazard incidents. A quarter of the primary PPH and a fifth of the concurrent PPH reports involved close others (*n* = 4, 25% and *n* = 13; 20%, respectively). Types of incidents that did not contain any PPH hazard were for example reporting that an ultrasound report was saved in the wrong patient record; or a delayed insulin administration.

Furthermore, we identified events that, on their own, could not be classified as being actually harmful and which could be rather considered as nuisances or unpleasant situations. However, we tagged and reviewed them and thus identified a class of events that could be expected to become potentially harmful when accumulating with other psychologically harmful events. We called them ‘cumulative’. In 20 (3.2%) reports, hazards for cumulative events were identified, such as unnecessary blood withdrawals, having to do unnecessary trips to the hospital, being given the wrong food, i.e. not respecting allergies, or unnecessary food intake restrictions.

## Discussion

In investigating incidents reported by cancer care personnel from three hospitals, we sought to gain insights about the extent to which hazards for primary PPH are reported by HCW within cancer care, as well as to categorize different types of PPH hazards. Due to how incident reporting systems are used in Switzerland, the analysed reports described many near-misses, and some potentially harmful events. However, negative patient outcomes or adverse events were not reported.

### Statement of principal findings

In roughly 15% of all analysed incident reports, hazards for psychological harm were identified, the vast majority of which consisted of accounts of concurrent PPH. i.e. a hazard for PPH that occurred together with a hazard for physical harm. The most frequently identified type of hazard in the small proportion of primary PPH (less than three percent) was informing patients or their close others inappropriately or contradictorily, e.g. about a subsequent intervention—a finding that is backed by prior research pointing to various harmful communication issues [11, 12]. Hazards such as privacy or autonomy violations and the failure to care for personal items [3, 6, 11], have also been reported before as psychologically harmful. The analysis furthermore revealed well-known safety hotspots as potential contributors to psychological harm, for example experiencing poor transitions either within or between institutions [8]. This finding underlines that poor organizational practices can significantly contribute to primary PPH.

The importance of organizational and managerial issues for preventing psychological harm was also illustrated in the examples of the hazard ‘errors potentially affecting the effectiveness of cancer care’: radiological and oncological departments not coordinating the care for a patient, or prescriptions not being fully administered. Two types of concurrent PPH hazards exemplify the detrimental effects of mismanaging a patient’s information, for example when critically ill patients have to interact with HCW who do not know about their diagnosis and treatment plans; or when patients have to inform HCW about relevant data that should be on record; or when patients are witnessing HCW cumbersomely collecting critical information.

Even though causes and organizational precursors for the identified hazards of psychological harm were not investigated, it is evident that a large variety of the incidents demonstrate hazards that do not require large resources to be eliminated or mitigated: for example, being sent to the right locations within a hospital, handing patients up-to-date treatment plans, or identifying the right patient before talking to them about a diagnosis or treatment.

The proportion of PPH reports mentioning close others [in 17 (18%) of the PPH-related incidents] points to an existing understanding that they are also affected by psychologically harmful events. While family involvement is often promoted as a resource for patient safety, this perspective risks obscuring a critical concern: the inappropriate shifting of safety-related responsibilities to close others, who may lack the necessary knowledge or resources to act effectively. When families are implicitly or explicitly expected to monitor care quality, coordinate tasks, or detect errors, they may find themselves in roles for which they are neither trained nor prepared. This role strain can result in overburdening, emotional exhaustion, and moral distress, potentially harming both the caregiver and the patient.

### Strengths and limitations and interpretation within the context of the wider literature

While this analysis revealed potential PPH associated with cancer care from the perspective of HCW, it provides only qualitative insights and does not assess the actual frequency or relative importance of the identified types of PPH. We believe that the reported number of PPH incidents likely underrepresents actual hazard prevalence due to several factors: reporters’ perceptions of what constitutes a safety issue may lead to significant events being ignored and therefore excluded from the analysis; as well as their underlying motivation to report, such as assigning blame or making other professional groups aware of issues, may lead to biases [20]. Furthermore, the breadth of issues covered in our analysis is limited, because only reports by HCW were examined. For instance, studies eliciting patients’ reports [8, 11, 12] bring up hazards that our analysis did not find, such as the potential harmfulness of prognostic discussions, or ‘not listening to the patient’ [12]. Still, the hospitals studied were typical providers of cancer care in Switzerland and therefore these findings should be generalizable.

Underreporting is a well-known phenomenon of IRS in healthcare institutions [14, 20]. Here, it may indicate a lower sensitivity among healthcare personnel in recognizing preventable psychological harm, potentially reflecting the traditional focus of patient safety activities. Preventable harm may still be understood as predominantly physical or closely related to it, as the higher percentage of concurrent PPH hazards may indicate. This is an important result, because perceiving and capturing harmful events is essential to prevent them. In a recent review investigating hazards and harm reported within incident reporting systems, psychological harm incidents were not categorized [21]. The WHO classification system for patient safety, even though mentioning that the harm caused by an incident maybe psychological, only has the incident type ‘behavior’ that may serve as an umbrella term for psychologically harmful events [22]. The policy guidance on recording patient safety events of the British National Health Service [23], however, now differentiates physical from psychological harm in their definitions, which is an important step to shed more light on how and how frequently patients are harmed psychologically. Future research should take this differentiation into account.

Events in IRS are limited in their interpretation due to various factors. The reports are motivated by observations or experiences of HCW and only describe a small fraction of the whole context of the situation. Additionally, the motivation of the reporters is not generally identifiable within the data. Our aim was hence to ensure that instances of potential psychological harm were accurately identified, regardless of the reporter’s motivation and using the information that was given. Due to these limitations, we could not examine whether PPH actually occurred, nor was its severity or potential consequences assessed. Even in psychologically harmful situations, individual responses vary widely, influenced by factors such as personality traits and available social support. Prior research has shown that psychologically harmful situations connected to end-of-life practices in intensive care units may lead to prolonged grief disorder, and symptoms of post-traumatic stress disorder in bereaved family members [24, 25]. These results illustrate that the consequences of harmful events may be severe and manifest with a time lag. The potential consequences of PPH events may harm individual patients or their close others to varying degrees, even if they were confronted with the same events. The perception of and resilience to psychological harm are individual and influenced by multiple contextual factors, such as the level of distress a patient experiences or the extent to which they feel socially supported [6]. As with physical harm events, acknowledging the event and offering honest regret and apology may help to limit the negative consequences of such events [26]. However, this requires awareness and perception of these events by HCW which—as our analysis suggests—remain largely invisible.

### Implications for policy, practice, and research

As this study shows that hazards for PPH are probably highly underrepresented in incident reports, future research should widen the perspective on PPH, thus identifying more potential types of events, as well as investigating the causes and consequences of PPH for patients, their close others, and for HCW. Triangulation of methods to detect PPH is an important avenue for future research. Our future research will survey patients and HCW to identify a larger variety of hazards for PPH and to explore its frequency, potential causes and consequences. Studying preventable psychological harm independent from physical harm is important, so that patient safety improvements addressing psychological harm can be designed and implemented, and awareness for it raised. The finding that consecutive minor psychologically harmful events may build up and result cumulatively in harm relates to similar effects observed in other domains: For example, ‘weathering’ [27] describes how accumulating stressful events from experiencing racism or poverty wear the body down and make people sicker over time. Similarly, HCW who are repeatedly exposed to patient deaths or placed in ethically distressing situations may, overtime, experience a cumulative emotional strain, including what is known as ‘cumulative grief’, which contributes to the development of burnout, compassion fatigue, or moral distress [28, 29]. Thus, investigating how accumulating harmful events, even if minor, may build up over time, and progressively have a significant impact represents a promising future research avenue.

## Conclusions

In summary, a variety of hazards for primary psychological harm occurring independently from physical harm were identified. While imminent physical harm was more prominent in the incident reports, few events involving ‘pure’, i.e. primary, psychological harm were reported. Many types of hazards for psychological harm appeared to be closely linked to organizational practices and are thus preventable with improvements of processes, structures, and work design. Future research and hazard and identification practices need to consider potential psychological harm to fully comprehend the level of patients’ safety. Although this study focused on cancer care, the findings may have broader implications for other medical specialties, particularly those serving seriously ill and vulnerable patients.

Author contributions

Yvonne Pfeiffer (Conceptualization, Data curation, Investigation, Methodology, Writing—original draft, Writing—review & editing), Lara Dreismann (Data curation, Investigation, Methodology, Writing—review & editing), Sofia C. Zambrano (Conceptualization, Funding acquisition, Investigation, Methodology, Resources, Supervision, Writing—review & editing), David L.B. Schwappach (Conceptualization, Funding acquisition, Investigation, Methodology, Resources, Supervision, Writing—review & editing)

Conflict of interest: None declared.

## Data Availability

The data analysed are institutionally owned and subject to confidentiality agreements. Therefore, the data are not publicly available.
